# Enhancement in physicochemical parameters and microbial populations of mushrooms as influenced by nano-coating treatments

**DOI:** 10.1038/s41598-021-87053-w

**Published:** 2021-04-12

**Authors:** Rokayya Sami, Abeer Elhakem, Amina Almushhin, Mona Alharbi, Manal Almatrafi, Nada Benajiba, Mohammad Fikry, Mahmoud Helal

**Affiliations:** 1 Department of Food Science and Nutrition, College of Sciences, Taif University, P.O. 11099, Taif, 21944 Saudi Arabia; 2 Department of Biology, College of Science and Humanities in Al-Kharj, Prince Sattam Bin Abdulaziz University, Al-Kharj, 11942 Saudi Arabia; 3 Department of Basic Health Sciences, Deanship of Preparatory Year, Princess Nourah Bint Abdulrahman University, P.O. Box 84428, Riyadh, 11671 Saudi Arabia; 4 Department of Agricultural and Biosystems Engineering, Faculty of Agriculture, Benha University, Moshtohor, Toukh, 13736 Qalyoubia Governorate Egypt; 5 Department of Mechanical Engineering, Faculty of Engineering, Taif University, P.O. 11099, Taif, 21944 Saudi Arabia

**Keywords:** Biochemistry, Biological techniques, Biotechnology, Nanoscience and technology

## Abstract

White button mushrooms are greatly high perishable and can deteriorate within a few days after harvesting due to physicomechanical damage, respiration, microbial growth of the delicate epidermal structure. For that reason, the present research work was applied to evaluate the effect of chitosan combination with nano-coating treatments on physicochemical parameters and microbial populations on button mushrooms at chilling storage. Nano coating with the addition of nisin 1% (CHSSN/M) established the minimum value for weight loss 12.18%, maintained firmness 11.55 N, and color index profile. Moreover, O_2_% rate of (CHSSN/M) mushrooms was the lowest at 1.78%; while the highest rate was reported for CO_2_ 24.88% compared to the untreated samples (Control/M) on day 12. Both pH and total soluble solid concentrations increased during storage. Results reported that the (CHSS/M) mushroom significantly (P < 0.05) reduced polyphenol oxidase activity (24.31 U mg^−1^ Protein) compared with (Control/M) mushrooms that increased faster than the treated samples. (CHSSN/M) treatment was the most efficient in the reduction of yeast and mold, aerobic plate microorganisms (5.27–5.10 log CFU/g), respectively. The results established that nano-coating film might delay the aging degree and accompany by marked prolongation of postharvest mushroom freshness.

## Introduction

Mushrooms *(Agaricus bisporus)* are common edible fungus that consumed globally due to their nutrients, bioactive compounds (*β-*glucan, tocopherol), protein, minerals, vitamins (B^group^, C), dietary fiber, and low in fats^1^. Besides, mushrooms may be a high-quality supplement for cereals and grains. White button mushroom is highly perishable as it has no outer skin to defend against the physicomechanical damage, respiration, microbial growth especially with *Pseudomonas* spp., enzymatic browning, or even water leakage^2^. Mushrooms are well known for their medicinal uses in constipation, neuroprotective, acidifying, antimicrobial and anti-inflammatory functions^3^. Zhaojun et al.^4^ established the shelf life of mushrooms is ranging from 3–4 days in the ambient temperature and an extra one day only leads the cap to be opened, color to be changed, and the stem to be elongated with a spongy and softy texture. Mushroom’s shelf-life is extremely limited for the distribution and the strategy of marketing due to the commercial losses. Consequently, several techniques were applied to prolong mushroom's shelf-life such as packaging, chilling, coating, nanocomposite films, washing with ascorbic acid, sodium chlorite, hydrogen peroxide, citric acid, and malic acid to act as antibrowning and antimicrobial resistance^5,6^. Chitosan is a non-toxic, natural food additive that is found in crustacean shells as shrimps and crabs which can prolong the shelf life due to effective antibacterial activity by forming a film around the fresh-cut produces or even the fruits as the whole bulk^7^. Nisin is well known for its antimicrobial activity as a safe food additive by (FAO/WHO) and can be applied in different food products^8^. Nanotechnology and the application of nanomaterials have been proposed as fillers to improve the packaging coating films. The use of silicon dioxide nanoparticles (1%) has been approved by the US Food and Drug Administration ^9,10^. Donglu et al.^11^ applied (Nano-PM) on mushrooms *(Flammulina velutipes)* which reduced the moisture loss, respiration rate, and elongation against the normal packing materials. Gholami et al.^12^ applied nanocomposite materials to prolong the shelf life of white mushrooms. Karimirad et al.^13^ applied chitosan nanoparticles and *Cuminum cyminum* oil for *Agaricus bisporus* shelf life extension.

As a result, the major object of the current research was to estimate the combined effect of chitosan, nisin, and silicon dioxide nanoparticles coating treatments on the shelf-life enhancement of whole button mushroom stored at 4 °C, investigating some physicochemical parameters and microbial populations to indicate the mushroom quality.

## Results

### Weight loss, firmness, and veil opening

**Figure 1:**
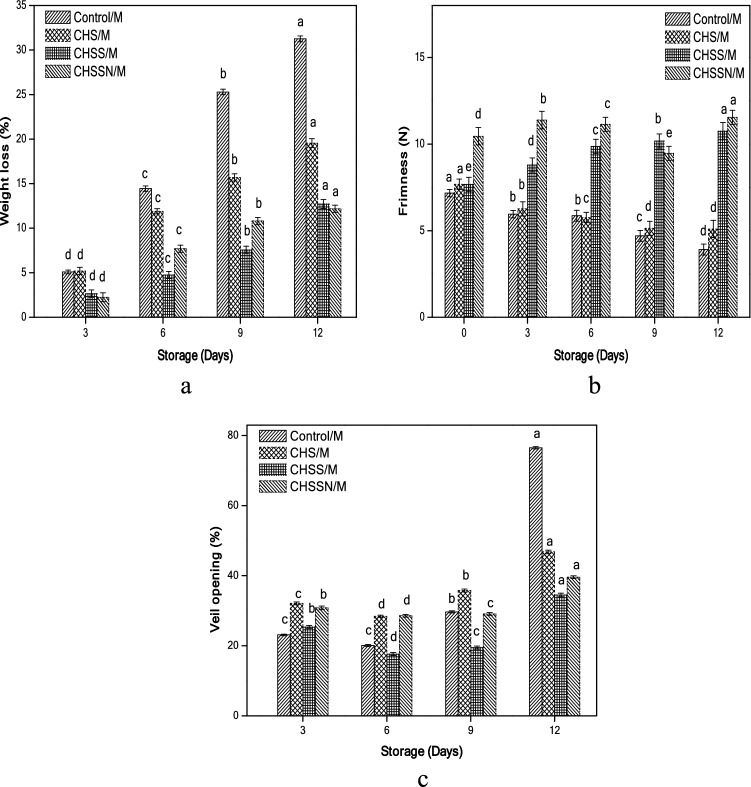
Effects of nano-coating treatments on weight loss (**a**) firmness (**b**), and veil opening (**c**).

The results of weight loss are shown in Fig. 1a shows that the maximum weight loss was detected on day 12 for (Control/M) ranging from 5.09 to 31.26% followed by chitosan mushrooms (CHS/M) 19.55%. The minimum value was established for chitosan/nano-silica/nisin mushrooms (CHSSN/M) 12.18% and chitosan/nano-silica mushrooms (CHSS/M) 12.72% mushrooms that can perhaps be due to nano-coating and the addition of nisin as it assists in retaining the respiration and regulating enzyme functions^14^.

As shown in Fig. 1b, the firmness observed a gradual decrease in all treatments and reached 3.91 N in (Control/M), whereas (CHS/M, CHSS/M, and CHSSN/M) reached (5.10, 10.75, and 11.55 N), respectively. Compared with the (Control/M) sample, (CHSSN/M) mushrooms reported a higher firmness value than other treatments during the storage. Figure 1c shows a higher percentage of veil opening in (Control/M) 76.51% as compared to the other chilled coated samples stored at 4 °C. (CHS/M) samples established higher veil opening on day 12 accounted for 46.80%, meanwhile, veil opening occurred for (CHSS/M) was the lowest 34.51%.

### Color index profile

**Figure 2:**
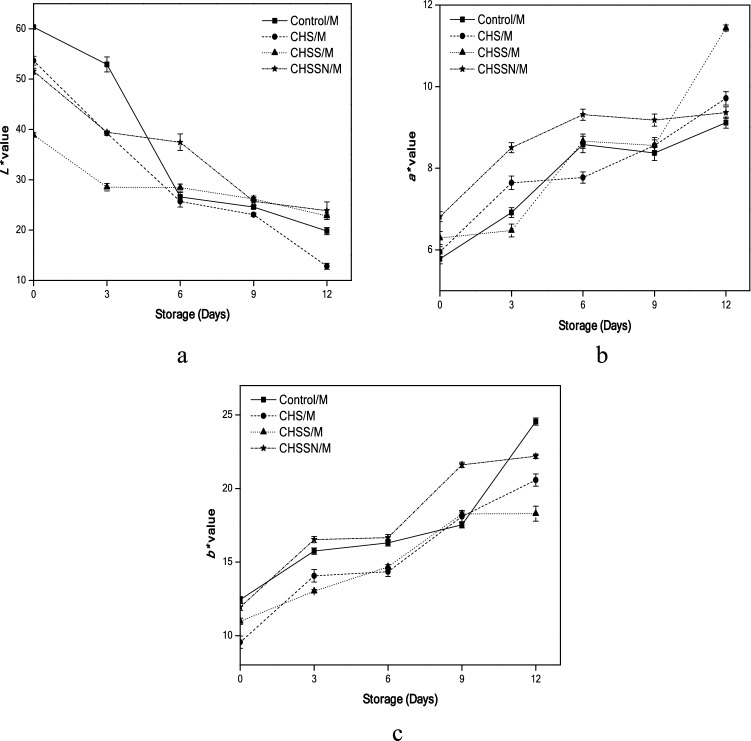
Effects of nano-coating treatments on color index profile L* (**a**), a* (**b**), and b* (**c**).

The whiteness index (L* value) is shown in Fig. 2a. As noted that (CHSSN/M) 23.86 coated mushrooms significantly increased the whiteness (p < 0.05) and exhibited the majority effect in delaying browning, followed by (CHSS/M) 22.84 compared with (Control/M) samples, on day 12.

a* value in (Control/M) samples obtained 5.78 on the 1st day, while (CHSS/M) mushrooms obtained the highest a* value 11.44 at the end of the storage period, which means changes occurred in the redness degree (Fig. 2b). b* value in (Control/M) samples obtained 12.44 on the 1st day, while (CHSSN/M) mushrooms obtained the highest b* value 22.19 at the end of the storage period, which means yellowness degree changes occurred (Fig. 2c).

The total color variation (ΔE) was calculated for (Control/M samples) from day 3 to 6 was significant amounting to 33.87 and 36.21, respectively. According to the results in (Table 1), all the coating treatments differed significantly against the initial mushrooms, due to high (ΔE) > 2. The smallest color differences were reported for (CHSSN/M) samples (ΔE from 36.00 to 37.94), while the highest color variation, (ΔE) = 10.58, was detected for (CHS/M) mushroom samples during the last days of the storage period.

**Table 1:** Changes of total color variation (ΔE) and browning index (BI).

Storage (days) at 4 °C	Control/M	CHS/M	CHSS/M	CHSSN/M
**ΔE**				
0	0^e^	7.30 ± 1.10^e^	21.53 ± 0.64^d^	8.89 ± 0.42^c^
3	8.26 ± 1.03^d^	21.24 ± 0.34^d^	31.84 ± 0.62^c^	21.46 ± 0.15^b^
6	33.87 ± 1.02^c^	34.51 ± 1.07^c^	31.85 ± 0.69^c^	23.32 ± 1.73^b^
9	36.21 ± 0.25^b^	37.81 ± 0.34^b^	34.78 ± 0.70^b^	36.00 ± 0.76^a^
12	42.44 ± 0.75^a^	48.39 ± 0.50^a^	38.39 ± 0.18^a^	37.94 ± 1.81^a^
**BI**				
0	29.37 ± 0.99^c^	27.04 ± 1.05^b^	43.98 ± 0.85^e^	35.20 ± 1.18^d^
3	43.85 ± 1.08^c^	57.25 ± 1.46^b^	75.37 ± 2.73^d^	68.25 ± 0.93^c^
6	112.39 ± 6.67^b^	99.67 ± 6.19^b^	91.57 ± 4.51^c^	75.09 ± 4.28^c^
9	136.46 ± 4.50^b^	157.32 ± 7.34^b^	131.90 ± 3.59^b^	172.97 ± 7.13^b^
12	363.83 ± 39.08^a^	775.22 ± 155.06^a^	170.34 ± 2.68^a^	208.26 ± 35.69^a^

*Results in a column followed by various uppercase letters indicate the significance (p < 0.05) analyzed by the test of Duncan’s multiple-range.

The browning index (BI) values of (Control/M) mushrooms increased from 29.37 to 363.83 after 12 days. Though, the BI value was the lowest 170.34 for (CHSS/M) mushrooms than the other coating treatments (Table 1).

### Headspace gas composition

**Figure 3:**
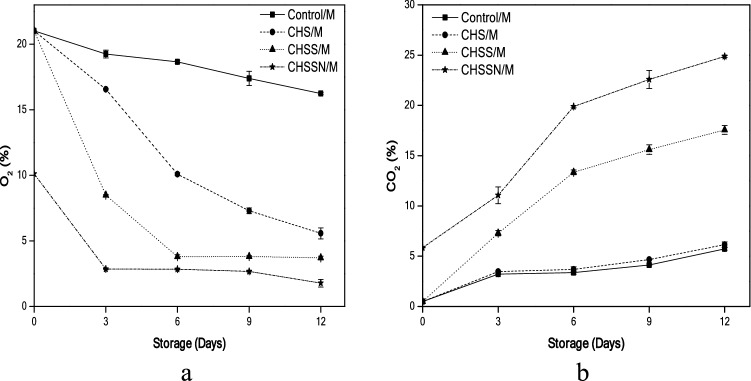
Effects of nano-coating treatments on headspace gas composition O_2_ (**a**) and CO_2_ (**b**).

O_2_ concentrations were influenced by various coating treatments are presented in (Fig. 3a). Noticeably, O_2_% rate of (CHSS/M) mushrooms 3.80% was reduced rapidly than (CHS/M) mushrooms 10.09% on the 6th day of the storage period. The final O_2_% rate of (CHSSN/M) mushrooms was the lowest 1.78 5% on day 12, which would efficiently avoid the anaerobic respiration^15^.

On the other hand, CO_2_ concentrations gradually raised during the whole storage period, (Fig. 3b). (CHSSN/M) mushroom samples established the highest rate 24.88% compared to (Control/M) samples, which could be due to the combined effect of high sample respiration and low carbon dioxide breakthrough of the coating treatment film. By contrast, (CHS/M) mushroom samples exhibited the lowest CO_2_ rate during the storage period.

### Active acidity and total soluble solids concentrations

**Figure 4:**
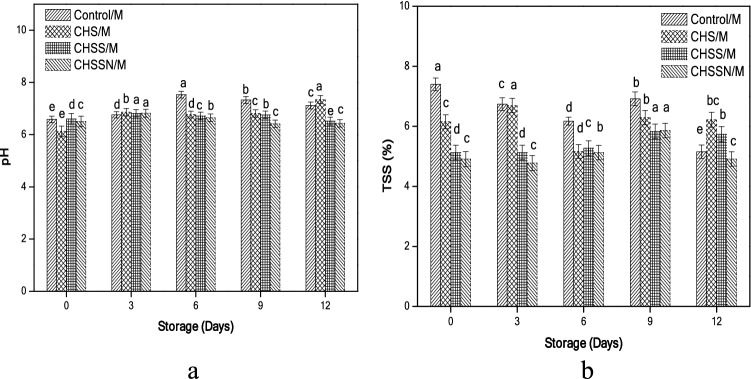
Effects of nano-coating treatments on active acidity (**a**) and total soluble solids concentrations (**b**).

Changes in acidity (pH) and total soluble solids (TSS) concentrations in button mushrooms are presented in (Fig. 4a,b). The initial pH value of all treatments was ranging from 6.13 to 6.61. The highest pH value on day 12 was established for (CHS/M) samples, while (CHSS/M and CHSSN/M) treatments established similar values as pH (6.52 and 6.43), respectively. (CHSSN/M) reported similar results for the total soluble solid concentrations.

### Membrane electrolyte leakage and PPO enzyme activity

**Figure 5:**
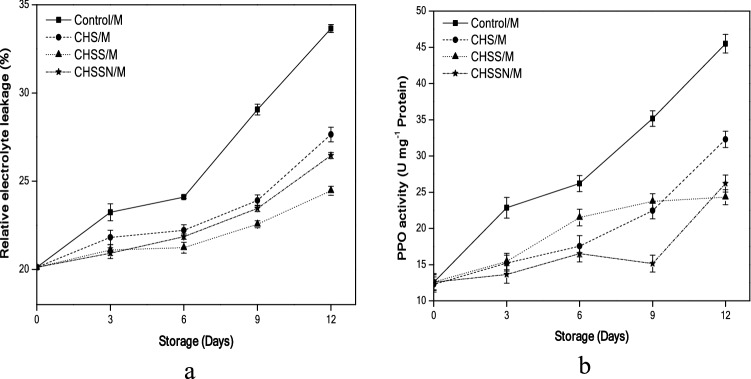
Effects of nano-coating treatments on membrane electrolyte leakage (**a**) and PPO enzyme activity (**b**).

According to (Fig. 5a), mushroom relative electrolyte leakage rates have been increased over the storage times.

(CHSS/M) mushroom samples 24.45% significantly (p < 0.05) decreased the relative leakage compared with (Control/M) samples 33.66% after 12 days of storage, while (CHSSN/M and CHS/M) recorded 26.45% and 27.64%, respectively.

Results reported that the (CHSS/M) mushroom significantly (P < 0.05) reduced the PPO activity (24.31 U mg^−1^ Protein) compared with (Control/M) mushrooms that increased faster than the treated samples, (Fig. 5b).

### Microbiological populations

**Figure 6:**
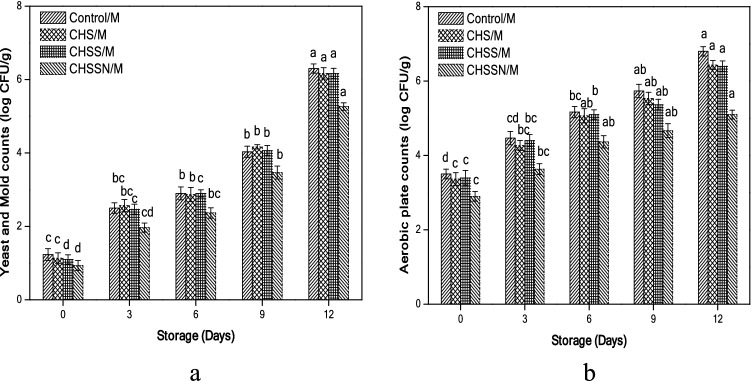
Effects of nano-coating treatments on microbiological populations, yeast and mold (**a**), aerobic plate counts (**b**).

Results are given in (Fig. 6a,b) reports that the initial total yeast and mold, aerobic plate colonies were reduced after applying different coating treatments. Total yeast and mold, aerobic plate counts of (Control/M) mushroom samples increased with the progress of the storage period at 4 °C (1.23–6.30 log CFU/g). In the current finding, (CHSSN/M) treatment was more efficient in inhibiting the growth of yeast and mold counts after 12 days of storage, as the count reached 5.27 log CFU/g.

Chitosan treatment (CHS/M) alone or even nanomaterial chitosan combinations (CHSS/M) detected equal values 6.17 log CFU/g as the results in the previous study^16^. Moreover, (CHSSN/M) treatment reduced the aerobic plate counts of 5.10 log CFU/g compared with (Control/M) samples 6.80 log CFU/g, which established dramatically browning spot contaminations on the cap.

In summary, the effect of chitosan combination with nano-coating treatments on physicochemical parameters and microbial populations of button mushrooms at chilling storage was examined. The results established that nano-coating with the addition of nisin 1% extended the mushroom shelf life of up to 12 days. Nano coating maintained nutrients, texture, lower respiration rate, weight loss, browning degree, and microbial contaminations. While CHSSN/M treatment reported the lowest PPO activity and CHS/M treatment preserved the lightness. Further research work is needed to evaluate more nanomaterials with the combination of antimicrobial agents for improving the quality of food products as novel packaging materials for other vegetables and fruits.

## Discussion

Normal moisture content in fresh mushrooms about 90% and dehydration can affect the appearance, economic demand, quality, and earlier senescence during postharvest storage^14,17^. The weight loss with percentage increase had also been established due to vapor transmission and respiration irrespective of whether the treatment or even coating materials^7,18^. Finding by Gholami et al.^12^, achieved similar results for firmness values after applying nanocomposite materials on white mushrooms. Firmness is a very vital quality parameter in evaluating mushroom's shelf-life with consideration to customer preference. The firmness decrease after harvesting can be due to cell growth, water migration, and the absence of a protective surface cuticle^3,19^. CHSSN/M treatment decreased the moisture loss and enhanced the firmness. Mushroom's veil opening is a result of a decrease in water cohesive forces and proteins for the intact condition of veils and caps. Besides, mushrooms continue growing after harvesting^20^. The results established a positive correlation between weight loss and veil opening percentage that indicated rising weight loss can cause rising in veil opening too. On the other hand, another negative correlation has been observed between firmness and veil opening which means when the veil opening raises, the firmness rate could reduce during the whole storage period. It is well known that white button mushrooms easily turn from white to brown color within only a few days of harvesting, which influences acceptance for customers, marketing, and processing. Enzyme activities and moisture loss can control the optical reduction as the coating treatments may cause changes on the surfaces of the mushrooms^12^. The finding is in agreement with Oz et al.^21^ who reported that color is influenced by enzyme oxidations, microbial population, senescence, and loss of nutrients. (ΔE) was > 2, which means a significant color difference. Besides, (ΔE) values > 5 the observer had two colors impression^22^. Qu et al.^23^ reported the browning was caused by the activity of PPO enzyme and microbial growth on the tissue. In conclusion, nano-coating and the addition of nisin prevent brown patches formation, repining delay, and maintaining color. Though, the decrease in O_2_ rates varied rapidly during the whole storage. High respiration and headspace gas composition can be influenced by nano coating^2,24^. The presence of nano-coating and the addition of nisin that had the lowest oxygen (O_2_) permeability, inhibited mushroom polyphenol oxidase activity, and respiration. Sami et al.^18^ reported the nanocomposite material effectively reduced O_2_ concentration consumptions on the effective oxygen barrier. Similar findings were reported for carbon dioxide composition as it can be a result of the mushroom softening^15^. Both pH and TSS concentrations increased during storage, in the same way to Jiang^25^. The pH can be influenced during the storage period due to the microbial growth rate^18^. These results may explain low respiration rates that reduce the synthesis and metabolites uses due to the slower hydrolysis of carbohydrates into sugar. The electrolyte leakage rate is well known as injury degree and it is one of the vital indexes for cell membranes semi-permeable characteristics, moreover, lipid peroxidation can lead to an increased rate of membrane integrity^26^. Qiao et al.^6^ reported that the combination between nano-films and the antimicrobial agents can control the electronegative for the permeability of cells. The results established that nano-coating film might delay the mushroom aging degree and accompany by marked prolongation of postharvest mushroom freshness. Ding et al.^27^ reported that electrolyte leakage reduction can be due to cellular contact decrease which can recover mushroom color. The mushroom membrane integrity is directly linked with the mushroom lipid peroxidation^27^. Enzyme is the major reason for white mushroom browning as it can catalyze the polyphenolic matrix to create dyes, which leads to a reduction in marketable values^6,18^. The results indicated that nano-coating films have a positive effect on delaying the enzymatic browning and a certain inhibitory effect on the activity of polyphenol oxidase of mushrooms. White button mushrooms might be exposed to microbial growth during processing and marketing. The reduction of the yeast and mold counts could be due to the presence of nisin as a good inhibitory effect for antimicrobial activity^8^. Microbial growth can be influenced by acidity and high sugar contents^19^. Nano-materials, due to their chemical and physical characterizes, have been used for developing some novel antimicrobial and antiviral agents^28–30^. These results reported that chitosan/nano-silicon dioxide and nisin 1% coating treatment was the most efficient in the reduction of yeast and mold, aerobic plate microorganisms in white button mushrooms.

## Materials and methods

### Materials

Chitosan (85%), nano-silicon dioxide (Purity > 99 wet%, 15 nm) and nisin were purchased from Sigma, MO, USA. The amount of the deposited solution was 450 mL.

### Methodology

Button mushroom samples were grown in the department of food science, Taif University, KSA, harvested in uniform size with caps range from 3.00 to 4.00 cm in diameter in the morning hours. Button mushrooms were free-damaged, deterioration, dirt attached to mushrooms was removed. (Control/M) were washed properly with distilled water and then air-dried.

### Films preparations

**Figure 7:**
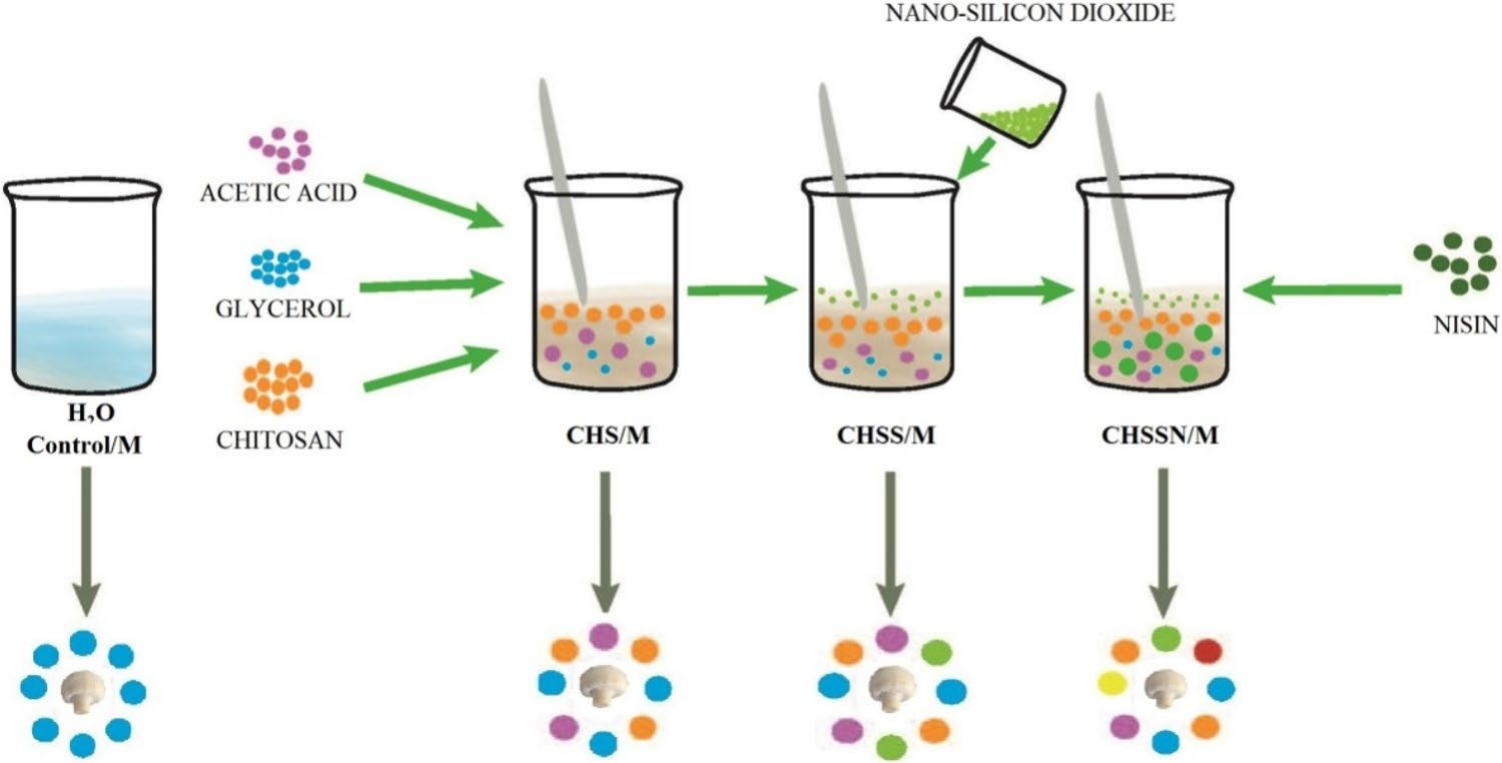
Films preparation.

The main coating film was prepared by mixing chitosan 1%, acetic acid 1% as a stabilizing agent, and glycerol 0.5% (CHS/M), (Fig. 7). The film was well-stirred overnight at 200 rpm with (pH 5.6) and then centrifuged at 4 °C. Approximately 1% of nano-silicon dioxide was added in a 1L container and sonicated 300 W at 60 °C (KQ-250 E, China) for 30 min, and named as (CHSS/M) and the other film was prepared by the addition of nisin 1% containing 0.02 mol/L hydrochloric acid to (CHSSN/M) as an antimicrobial agent and renamed as (CHSSN/M).

### Sample treatments

The whole samples were dipped in coating solutions for 15 min, placed on a trellis shelf to allow to lose the extra moisture followed by packaging in a zipped lock (40 mm) thickness polyethylene bags with twelve perforations (diameter hole: 5 mm)^31^. The coated and uncoated mushroom samples were chilled at 4 °C with a relative humidity of (80–85%). All the physicochemical parameters and microbial populations were estimated at an interval of 3 days and carried out up to 12 days.

### Weight loss, firmness, and veil opening

The weight loss of the mushroom samples was measured as the percentage (%) of the initial and final weights until the end of the storage experiment^25^.

1 $$\mathrm{Weight loss}\left(\mathrm{\%}\right)=\left(\frac{{\mathrm{W}}_{0}-{\mathrm{W}}_{\mathrm{t}}}{{\mathrm{W}}_{\mathrm{t}}}\right)\times 100$$

where W_0_ is the initial weight and W_t_ is the weight at the different times during the storage period.

The firmness was recorded in Newton (N) and evaluated by a TMS-PRO texture analyzer (Food Technology Co., USA) punched with a 6 mm diameter and 3 mm depth with a crosshead speed of 20 mm min^−1^^32^. Veil opening was measured on each package was estimated visually based on the broken or cracked mushroom's volva and calculating the number of opened veils as a percentage of the whole bulk^33^.

2 $$Veil \, opening \, percentage \, \left( \% \right)\, = \,\left[ {\left( {{V_t}\, - \,{V_f}/ \, {V_i}} \right)\, \times \,100\% } \right]$$

(Where) V_t_ = Total numbers and V_f_ = Number of the open mushroom veils.

### Color index profile

The color index profile was evaluated by a CR-400 (Konica Co., Japan) for L*, a*, and b* values at three fixed locations on the mushroom caps^34,35^. The total color difference (ΔE) is reported by Eq. (3):

3 $$\Delta E = \, {\left[ {\left( {L* \, - \, L0} \right) \, 2 \, + \, \left( {a* \, - \, a0} \right)2 \, + \, \left( {b* \, - \, b0} \right) \, 2} \right]^{0.5}}$$

(where) L*0, a*0, and b*0 are the values of control samples at day 0. The browning index (BI), establishes the purity of the brown color, is reported by Eq. (4):

4 $$\begin{gathered} BI\, = \,[(100 \, \left( {x - 0.31} \right)]/ \, 0.172 \hfill \\ \;\left( {where} \right) \, x \, = \, (a * + \, 1.75L * )/(5.645L * + \, a * - \, 3.012b * ) \hfill \\ \end{gathered}$$

### Headspace gas composition

The analysis of CO_2_ (%) and O_2_ (%) concentrations in the coated samples were estimated in triplicate by a SCY-2 A O_2_/CO_2_ (Xinrui, China). For estimating the air composition with a 20 mL syringein on the outer surface of the mushroom samples and gas concentrations were expressed in percentage every 3 days (for 12 days)^32^.

### Determination of active acidity and total soluble solids concentrations

Active acidity pH was determined by homogenizing (15 g) of mushroom samples, centrifuging at 5000 rpm for 20 min, and extracting by Hanna HI 221 pH-meter^36^. The total soluble solid concentrations were detected from the centrifuged mushroom extracts with the help of the ATAGO PAL-1 refractometer (USA, Inc.) according to PN-90/A-75101/02^37^.

### Membrane permeability

Membrane permeability was detected with the help of electrolyte leakage. Approximately (5 g) of samples were sliced into discs 3 mm thick and 3 mm in diameter. These slices were submerged in 50 ml of distilled water for 1 h, dried, and suspended in 50 mL of deionized water. The initial electrical conductivity (C1) was detected by a conductivity meter (DDB-303A, China). The final electrical conductivity (C2) was detected after boiling for 30 min and cooling to the ambient temperature^23^. The electrolyte leakage rate was described by Eq. (5):

5 $$Leakage \, rate\left( \% \right)C1 \, / \, C2 \, \times \, 100.$$

### Determination of polyphenol oxidase (PPO) enzyme activity

Mushroom extracts were prepared by homogenizing 5 g of each coating treatment in 12 ml of phosphate buffer (pH 7) to (2.2 mol/L). PPO activity was determined by incubating 0.5 ml of enzyme extracts in 2.5 ml of sodium phosphate buffer solution and 0.5 ml catechol (0.1 M) as a substrate, then centrifuging at 1000 rpm for 15 min at 4 °C. Values were detected at 398 nm and expressed as U mg^−1^ protein^13^.

### Microbiological analysis

For microbial analysis, yeast and mold counts, aerobic plate counts in all coating treatments were achieved *in-vivo* and detected during chilling storage at 4 °C. Thirty grams of mushroom samples were homogenized with 225 ml of (0.1%) peptone water for 5 min^32^. Serial dilutions (10^−1^, 10^−2^, and 10^−3^) were plated on potato dextrose agar and incubated at 28 °C for 7 days to evaluate yeast and mold counts. Total aerobic plate counts were evaluated on plate count agar and incubated at (35 °C for 2 days and 4 °C for 7 days), respectively. The microbial analysis was evaluated and expressed as log CFU/g.

### Statistical analysis

All the experiments were applied in triplicates (3) and results were analyzed by using variance (ANOVA) which considers various nano-coating treatments at (P ≤ 0.05) by using SAS 9.4 version.
